# Data for interventional role of training in changing the knowledge and attitudes of urban mothers towards food hygiene (A case study of Ravansar Township, Kermanshah, Iran)

**DOI:** 10.1016/j.dib.2018.05.021

**Published:** 2018-05-18

**Authors:** Yahya Safari, Sara Maleki, Kamaleddin Karimyan, Hossein Arfaeinia, Vinod Kumar Gupta, Nasrin Yoosefpour, Naseh Shalyari, Maliheh Akhlaghi, Hooshmand Sharfi, Arash Ziapour

**Affiliations:** a Research Center for Environmental Determinants of Health (RCEDH), Kermanshah University of Medical Sciences, Kermanshah, Iran; b Department of Public Management, Faculty of Management and Accounting, Allameh Tabataba'i University (ATU), Tehran, Iran; c Environmental Health Research Center, Kurdistan University of Medical Sciences, Sanandaj, Iran; d Department of Environmental Health Engineering, Faculty of Public Health, Bushehr University of Medical Sciences, Bushehr, Iran; e Department of Applied Chemistry, University of Johannesburg, Johannesburg, South Africa; f Department of Environmental Health Engineering, Faculty of Public Health, Tehran University of Medical Sciences, Tehran, Iran; g Student of Environmental Health Engineering, Health Faculty, Students Research Committee, Gonabad University of Medical Sciences, Gonabad, Iran; h Students Research Committee, School of Public Health, Kermanshah University of Medical Sciences, Kermanshah, Iran

**Keywords:** Food hygiene, Training, Urban mothers, Knowledge, Attitude

## Abstract

Food hygiene is a key factor at the time of production and distribution of food. Therefore, the present study aimed to assess the interventional role of education in changing the knowledge and attitudes of urbane mothers towards food hygiene in Ravansar Township, Kermanshah, Iran. To this end, 200 mothers residing in Ravansar Township were selected using simple random sampling. First, the subjects’ knowledge and attitudes towards food hygiene were evaluated in a pre-test, and then after holding some educational sessions, the two variables were assessed again in a post-test using a researcher-made questionnaire with 72 questions. The reliability and validity of the questionnaire were evaluated using Cronbach's alpha and content validity, respectively. After completing the questionnaires, the results were analyzed using the SPSS Statistical Software Version 21.0, and all tests were at the significance level of *α* = 0.05. The results of the present study demonstrated that education did not promote the knowledge of married subjects, those whose use of media was average or high, and the ones aged above 20 (*P* > 0.05). However, the results showed that education had significant effects on other factors (*P* < 0.05). In addition, it was revealed that the effects of education on promoting the attitudes of individuals aged above 60, those holding academic education and married subjects were not significant (*P* > 0.05), Nevertheless, the results revealed that education had significant effects on other factors (*P* < 0.05). Hence, it can be concluded that education plays a major role in changing the knowledge and attitudes of urban mothers towards food hygiene.

**Specifications Table**

**Table:** 

Subject area	Environmental sciences
More specific subject area	Health sciences
Type of data	Tables
How data was acquired	To do the present research, 200 mothers residing in Ravansar Township were selected using simple random sampling. First, the subjects’ knowledge and attitudes towards food hygiene were evaluated in a pre-test, and then after holding some educational sessions, the two variables were assessed again in a post-test using a researcher-made questionnaire with 72 questions. After completing the questionnaires, the results were analyzed using the SPSS Statistical Software Version 21.0, and all tests were at the significance level of *α* = 0.05.
Data format	Raw, analyzed
Experimental factors	The reliability and validity of the questionnaire were evaluated using Cronbach's alpha and content validity, respectively.
Experimental features	To compare the means of two groups of variables and more, the independent sample t-test and ANOVA were used, respectively. In addition, the role of education in changing the knowledge and attitudes of interviewees was evaluated using paired t-test.
Data source location	Ravansar Township, Kermanshah, Iran
Data accessibility	Data are included in this article

**Value of the data**

• Based on the results of previous studies, proper education can play major roles in promoting knowledge and attitudes of individuals towards health issues, especially food hygiene [1], [2], [3], [4], [5]. Hence, the data from the present study provides a background to the above-mentioned goal, especially in food safety.
• Proper education can cause changes in people's knowledge and attitude. The data of this study emphasizes the above mentioned subject in the field of food hygiene [2], [3].
• So far, no previous studies have been conducted on the subject under study in Ravansar Township, or even in Kermanshah Province. So, the obtained data of the present study can be useful for both similar future studies and educating the mothers residing in Ravansar Township about food hygiene.
• The data of the present study can provide the requirements for planning the education of mothers and housewives on healthy food.
• The data of present study showed that proper education can have important and positive effects on the promotion of urban mothers’ knowledge and attitudes towards food hygiene.

## 1 Data

The present study aimed to assess the interventional role of education in changing the knowledge and attitudes of urbane mothers towards food hygiene in Ravansar Township, Kermanshah, Iran in 2017. The results showed that 38 subjects (19%) were single and 182 persons (81%) were married, and according to the interviewees, 91% used media (radio, TV and newspaper) covering food hygiene topics. In addition, 82% of the interviewees said that their use of media about foodstuffs was at average to low levels. Based on the results of the present study, only 24% of the subjects held academic education, and the majority of subjects were in the 40–60 age group (see Table 1, Table 2).

**Table 1:** The total scores obtained by the subjects on knowledge of food hygiene (before and after training) based on the variables under study.

**Variables**		**Frequency**		**Before training**		**After training**		***P*****(to compare before and after training)**
		***N***	**%**	**Mean ± SD**	***P*****(between groups)**	**Mean ± SD**	***P*****(between groups)**	
Marital status	Divorced	18	9	16.96 ± 2.4	0.014	20.32 ± 1.8	0.061	0.021
	Married	182	91	22.12 ± 3.41		25.11 ± 2.45		0.036
Type of area in terms of welfare	Rich	100	50	22.23 ± 2.9	0.005	25.10 ± 3.2	0.042	0.035
	Poor	100	50	18.13 ± 2.55		22.43 ± 4.34		0.014
Whether, use the media (radio, TV, newspapers, and magazines) in relation to food hygiene issues?	Yes	182	91	22.1 ± 2.22	0.007	25.15 ± 3.34	0.039	0.045
	No	18	9	17.23 ± 1.98		21.41 ± 2.76		0.036
The use rate of the media in relation to food hygiene issues?	Not at all	18	9	16.15 ± 3.6	0.002	23.81 ± 1.9	0.35	0.005
	Low	22	11	23.7 ± 2.42		23.5 ± 2.4		0.013
	Medium	144	72	22.14 ± 4.23		24.44 ± 2.27		0.052
	High	16	8	25.47 ± 1.17		24.14 ± 1.75		0.491
Education level	Elementary	72	36	18.12 ± 2.91	0.009	23.14 ± 3.46	0.561	0.023
	Secondary education	52	26	18.44 ± 2.64		24.59 ± 2.93		0.035
	Diploma	28	14	18.56 ± 3.20		23.18 ± 4.54		0.037
	University education	48	24	21.90 ± 2.55		24.12 ± 2.63		0.049
Age group (year)	1–20	30	15	19.14 ± 3.43	0.026	23.55 ± 2.36	0.04	0.0451
	21–40	122	61	24.34 ± 5.1		26.18 ± 3.3		0.85
	41–60	34	17	21.4 ± 3.34		23.64 ± 3.54		0.144
	> 60	14	7	17.54 ± 2.7		18.87 ± 1.6		0.238

**Table 2:** The total scores obtained by the subjects on attitude towards food hygiene (before and after training) based on the variables under study.

**Variables**		**Frequency**		**Before training**		**After training**		***P*****(to compare before and after training)**
		***N***	**%**	**Mean ± SD**	***P*****(between groups)**	**Mean ± SD**	***P*****(between groups)**	
Marital status	Single	18	9	81.32 ± 8.12	0.039	91.31 ± 9.8	0.304	0.013
	Married	182	91	90.56 ± 6.92		92.72 ± 9.5		0.23
Type of area in terms of welfare	Rich	100	50	88.96 ± 9.16	0.025	93.96 ± 9.16	0.15	0.039
	Poor	100	50	67.66 ± 5.86		90.66 ± 5.86		0.002
Whether, use the media (radio, TV, newspapers, and magazines) in relation to food hygiene issues?	Yes	182	91	79.64 ± 9.15	0.179	92.7 ± 8.2	0.092	0.012
	No	18	9	80.71 ± 9.21		88.43 ± 9.32		0.041
The use rate of the media in relation to food hygiene issues?	Not at all	18	9	80.71 ± 9.21	0.11	90.4 ± 12.69	0.122	0.023
	Low	22	11	82.15 ± 7.13		90.4 ± 12.69		0.04
	Medium	144	72	84.9 ± 6.2		92.23 ± 7.9		0.022
	High	16	8	85.45 ± 7.35		93.75 ± 7.22		0.012
Education level	Elementary	72	36	80.45 ± 10.12	0.032	91.55 ± 8.22	0.251	0.011
	Secondary education	52	26	87.85 ± 6.65		93.15 ± 9.87		0.007
	Diploma	28	14	88.29 ± 6.9		92.2 ± 8.4		0.036
	University education	48	24	92.66 ± 5.53		93.23 ± 7.55		0.112
Age group (year)	1–20	30	15	80.92 ± 8.14	0.097	92.92 ± 8.44	0.073	0.034
	21–40	122	61	84.75 ± 9.48		92.22 ± 8.18		0.029
	41–60	34	17	83.33 ± 6.57		92.15 ± 6.57		0.041
	> 60	14	7	87.1 ± 2.2		86.34 ± 2.2		0.066

Further, the results indicated that the mean score of subjects’ knowledge about the variables under study before training was statistically significant (*P* < 0.05). The mean score of subjects’ knowledge prior to training was statistically significant among the relevant groups of variables (*P* < 0.05). However, in the post-training stage, this significance was not observed in variables such as use of media, education, and marital status (*P* > 0.05) (see Table 1).

While the mean score of attitudes in the pre-training stage was not significant among the groups related to the variables of level and status of using media and age group (*P* > 0.05). Not to mention, in the post-training stage, there was no significant difference in terms of each of the variables (*P* > 0.05) (see Table 2).

It was shown that education did not play effective roles in the knowledge of married subjects and those using media about food hygiene at average or high levels and those aged above 20 (*P* > 0.05). However, the results showed that education had significant effects on other factors (*P* < 0.05) (see Table 3). In addition, it was revealed that the effects of education on promoting the attitudes of individuals aged above 60, those holding academic education and married subjects were not significant (*P* > 0.05), Nevertheless, the results revealed that education had significant effects on other factors (*P* < 0.05) (see Table 2).

**Table 3:** The subjects’ knowledge of food hygiene and safety in relation to each item based on the obtained scores.

**Number of component**	**Components**	**The knowledge level in each component based on the score**	
		**Before training (achievable maximum score)**	**After training (achievable maximum score)**
1	Knowledge about food contamination, causes, side effects, and diseases associated with it	5.75(8)	6.9(8)
2	Knowledge about healthy food and simple way to identify it’s	5.10(11)	8.54(11)
3	Knowledge about correct way of food storage, with aim of provides its health	3.98(7)	6.15(7)
4	Knowledge about the correct way of cooking food and health requirements while cooking	2.53(5)	4.2(5)
5	Knowledge about the correct way of fruits and vegetables disinfection	1.03(2)	1.44(2)

The results of the present study demonstrated that the subjects’ knowledge of the first (attitudes towards the importance of hygienic foodstuffs and its simple identification) and fourth components (knowledge about proper cooking and observing hygienic rules during cooking) was at good levels. However, the subjects’ knowledge of the said component was promoted to a very good level after education (see Table 3). In addition, the results indicated that the subjects’ attitudes towards evaluation was desirable only for the second component, i.e. attitudes towards the importance of hygienic foodstuffs and its simple identification. However, the attitude was at a desirable level in other special aims (see Table 4).

**Table 4:** The subjects’ attitude of food hygiene and safety in relation to each item based on the obtained scores.

**Number of component**	**Components**	**The knowledge level in each component based on the score**	
		**Before training (achievable maximum score)**	**After training (achievable maximum score)**
1	Attitude about food contamination, causes, side effects and diseases associated with i	28.8(39)	36.6(39)
2	Attitude about healthy food and simple way to identify it’s	19.5(24)	21.92(24)
3	Attitude about correct way of food storage, with aim of provides its health	11.48(15)	14.32(15)
4	Attitude about the correct way of cooking food and health requirements while cooking	3.18(6)	4.71(6)
5	Attitude about the correct way of fruits and vegetables disinfection	3.75(5)	5.46(6)
6	Attitude about the importance individual health in food hygiene	6.79(9)	8.27(9)

## 2 Study design, materials and methods

To carry out the present experimental study, a researcher-made questionnaire was first designed using the basic principle of food hygiene presented in national and international books and articles [6], [7], [8], [9], [10], [11], [12], [13], [14], [15], [16], [17]. The questionnaire consisted of 72 questions: six questions on demographic information (education, age group, living area, marital status, using media etc.), 33 questions on knowledge, and 33 questions on attitudes. The knowledge and attitude questions were designed based on five and six components, respectively (see Table 5, Table 6). Additionally, the levels of knowledge and attitudes of the population under study were divided into four subscales ("poor", "average", "good", and "very good") with specific scores in each subscale (see Table 5, Table 6).

**Table 5:** The rankings of knowledge for each of the components under study based on Likert scale.

**Number of component**	**Components**	**The number of questions covering the components**	**Achievable maximum score**	**Knowledge level**			
				**Weak**	**Medium**	**Good**	**Very good**
1	Knowledge about food contamination, causes, side effects, and diseases associated with it	8	8	0–1.99	2–3.99	4–5.99	6–8
2	Knowledge about healthy food and simple way to identify it’s	11	11	0–2.74	2.79–5.49	5.5–8.24	8.25–11
3	Knowledge about correct way of food storage, with aim of provides its health	7	7	0–1.74	1.75–3.49	3.5–5.24	5.25–7
4	Knowledge about the correct way of cooking food and health requirements while cooking	5	7	0–1.24	1.25–2.49	2.5–3.74	3.75–5
5	Knowledge about the correct way of fruits and vegetables disinfection	2	2	0–0.49	0.5–0.99	1–1.49	1.5–2
**Overall knowledge**		33	33	0–8.24	8.25–16.49	16.5–24.74	24.75–33

**Table 6:** The rankings of attitude for each of the components under study based on Likert scale.

**Number of component**	**Components**	**The number of questions covering the components**	**Achievable maximum score**	**Attitude level**			
				**Weak**	**Medium**	**Good**	**Very good**
1	Attitude about food contamination, causes, side effects and diseases associated with it	13	39	0–9.74	9.75–19.5	19.5–29.24	29.25–39
2	Attitude about healthy food and simple way to identify it’s	8	24	0–5.59	6–12	12–17.99	18–24
3	Attitude about correct way of food storage, with aim of provides its health	5	15	0–4.49	4.5–9	9–13.49	13.5–18
4	Attitude about the correct way of cooking food and health requirements while cooking	2	6	0–1.49	1.5–2.99	3–4.49	4.5–6
5	Attitude about the correct way of fruits and vegetables disinfection	2	6	0–1.49	1.5–2.99	3–4.49	4.5–6
6	Attitude about the importance individual health in food hygiene	3	9	0–2.24	2.25–4.49	4.5–6.24	6.75–9
**Overall attitude**		33	99	0–24.74	24.75–49.49	49.5–74.24	74.25–99

The validity of the questionnaire was evaluated using content validity [18], [19], [20], [21], [22], [23]. To do so, the intended questionnaire was given to 10 faculty members of the Faculty of Health and 10 employees at the environmental health centers of Ravansar Township to be examined based on the objectives of the study and the questions relating to attitude and knowledge. Furthermore, the reliability of the questionnaire was evaluated using Cronbach's alpha (*α* = 0.86) [24], [25], [26], [27], [28], [29], [30]. According to the high level of this coefficient in comparison with 0.7, the internal correlation of the questions was confirmed. To do the present research, 200 mothers residing in Ravansar Township were selected using simple random sampling. Out of the 200 selected subjects, two groups of 100 were selected from the region with and without access to welfare facilities. The criteria for the said facilities included relative income, distance from/proximity to the main urban facilities (hospitals, schools etc.). Not to mention, in each group of 100 subjects, 40 had primary and elementary education, 30 secondary education, and 30 others had collegiate education. After performing the pre-test (completing the questionnaires in the first stage), the interviewees were provided with face-to-face training and educational pamphlets. Then, the questionnaires were completed after the training (post-test). Moreover, the contents of the educational pamphlets and lessons were evaluated and verified by experienced and expert faculty members who examined the reliability of the questionnaire, and their remarks were included.

After completing the knowledge and attitude questionnaires in two steps (pre-test and post-test), the results were transferred to the SPSS Statistical Software Version 21.0. To compare the means of two groups of variables and more, the independent sample *t*-test and ANOVA were used, respectively. In addition, the role of education in changing the knowledge and attitudes of interviewees was evaluated using paired *t*-test. Furthermore, all tests were at the significance level of *α* = 0.05.
