# Supplementary material for: Data for interventional role of training in changing the knowledge and attitudes of urban mothers towards food hygiene (A case study of Ravansar Township, Kermanshah, Iran)
Source: Data Brief. 2018 May 18;19:67–75. doi: 10.1016/j.dib.2018.05.021 (PMC5993158; doi:10.1016/j.dib.2018.05.021)
Supplement: Supplementary file 1 — Transparency document [file mmc1.doc]

Dear editor(s)

Data in Brief journal

I would be grateful if you would consider the attached manuscript “**Data for interventional role of training in changing the knowledge and attitudes of urban mothers towards food hygiene (A case study of Ravansar Township, Kermanshah, Iran)”** for publication in valuable Journal Data in Brief. We declare that this manuscript, or part of it, has neither been published nor is currently under consideration for publication by any other journal. We also declare that we have read the manuscript and approved submission to Journal of Data in Brief. The research was supported by Kermanshah University of Medical Sciences, Kermanshah, Iran. **None of the authors of this paper have any conflicts of interest to declare.**

Sincerely yours,

Corresponding author

Arash Ziapour
